# Reflex Convulsions

**Published:** 1897-06

**Authors:** Eric Vondergoltz

**Affiliations:** New York


### REFLEX CONVULSIONS.

ERIC VONDERGOLTZ, M. D., NEW YORK.

Case No. 454, February 3d, 1897.—E. N., thirteen years old; has been sick for three months.

Convulsions every three weeks; had them three times in last week.

Sudden unconsciousness. Face red. Frothing and blood from mouth. Sleeps after convulsion all day. Always occur in the morning. Headache, supra-orbital and occipital. High grade of nervousness.

Menses began three years ago; menses occur now every three weeks.

Feels dizzy. Constipation. Appetite good. Sleeps well.

The patient was under allopathic treatment till last week without any result, rather the last medicine had aggravated the general symptoms. Nux-vom. 30 (one dose).

February 10th—Somewhat better. S.-l.

February 13th—Patient had convulsions again. Patient was ordered to come to my office for examination.

February 18th—Examination. Vaginitis granulosa; uterus anteflected; cervix open; ovaries normal, left one prolapsed and sensitive on touch; intra-uterine examination easy, two and three-quarter inches deep cavity; hymen open, so that the examination was painless; external genitals anemic and livid; skin of lower abdomen and legs appeared pale and relaxed.

Urine contains too many phosphates, otherwise normal.

Patient seemed to me to be addicted to masturbation. Hyoscyamus 1000 (one dose).

February 20th—No headaches now; all nervousness gone. S.-l.

February 24th—Feels well. S.-l.

March 17th—Feels well. S.-l.

April 3d—Perfectly well. S.-l.

Patient discharged cured; will return so soon any symp toms are reappearing.

June 4th, 1897, was at my office, and reported herself perfectly well.

